# Effect of Camphorquinone Concentration in Physical-Mechanical Properties of Experimental Flowable Resin Composites

**DOI:** 10.1155/2018/7921247

**Published:** 2018-05-22

**Authors:** Dayany da Silva Alves Maciel, Arnaldo Bonfim Caires-Filho, Marta Fernandez-Garcia, Camillo Anauate-Netto, Roberta Caroline Bruschi Alonso

**Affiliations:** ^1^Instituto de Ciências Ambientais, Químicas e Farmacêuticas, Universidade Federal de São Paulo, Diadema, SP, Brazil; ^2^Universidade Anhanguera de São Paulo (UNIAN), São Paulo, SP, Brazil; ^3^Instituto de Ciencia y Tecnologia de Polimeros (ICTP-CSIC), Madrid, Spain; ^4^Universidade Metropolitana de Santos (UNIMES), Santos, SP, Brazil; ^5^Núcleo de Pesquisas Tecnológicas (NPT), Universidade de Mogi das Cruzes (UMC), Mogi das Cruzes, SP, Brazil

## Abstract

The aim of this study was to evaluate the effect of camphorquinone concentration in physical-mechanical properties of experimental flowable composites in order to find the concentration that results in maximum conversion, balanced mechanical strength, and minimum shrinkage stress. Model composites based on BISGMA/TEGDMA with 70% wt filler loading were prepared containing different concentrations of camphorquinone (CQ) on resin matrix (0.25%, 0.50%, 1%, 1.50%, and 2% by weight). Degree of conversion was determined by FTIR. Surface hardness was assessed before and after 24 h ethanol storage and softening rate was determined. Depth of cure was determined by Knoop hardness evaluation at different depths. Color was assessed by reflectance spectrophotometer, employing the CIE-Lab system. Flexural strength and elastic modulus were determined by a three-point bending test. Shrinkage stress was determined in a Universal Testing Machine in a high compliance system. Data were submitted to ANOVA and Tukey's test (*α* = 0.05). The increase in CQ concentration caused a significant increase on flexural strength and luminosity of composites. Surface hardness was not affected by the concentration of CQ. Composite containing 0.25% wt CQ showed lower elastic modulus and shrinkage stress when compared to others. Depth of cure was 3 mm for composite containing 1% CQ and 2 mm for the other tested composites. Degree of conversion was inversely correlated with softening rate and directly correlated with elastic modulus and shrinkage stress. In conclusion, CQ concentration affects polymerization characteristics and mechanical strength of composites. The concentration of CQ in flowable composite for optimized polymerization and properties was 1% wt of the resin matrix, which allows adequate balance among degree of conversion, depth of cure, mechanical properties, and color characteristics of these materials.

## 1. Introduction

Flowable resin composites are versatile materials with optimized handling characteristics that have been used in various aesthetic dental procedures, such as preventive restorations (for minimally invasive Class I and II); pit and fissure sealants; cavity liners; restoration of Class V abfraction lesions; bonding of orthodontic brackets; splinting fractured and mobile teeth (posttrauma or periodontal involvement); reattachment of fractured anterior tooth segment; repair of margins of crowns and restorations; bonding of fibre posts in the restoration of endodontically treated teeth [1–3]; even for higher Class II restorations, these composites have been successfully used [4].

Flowable composites differ from conventional restorative composites due to their reduced filler content, which permits a more intimal adaptation to the cavity walls, greater flow, and flexibility [2, 5–9]. Due to their great range of possible clinical applications, flowable composites must balance their properties to achieve adequate color match for aesthetic applications, great depth of cure, adequate conversion, low shrinkage stress for application in deep and high C-factor cavities (when used as restorative or liner material), flexibility and balanced mechanical properties to restore Class V abfraction cavities [1, 6].

To achieve satisfactory and balanced properties, the composition is determinant. A stable resin matrix would allow sufficient mechanical properties to resist the chemomechanical challenges of the oral function [10, 11]. A stable resin matrix can be obtained not only by a balanced combination of high and low molecular weight monomers [12], but also by an adequate and adjusted polymerization initiator system [10, 11, 13]. In this way, it is reasonable to assume that the initiator system, in the last instance, could affect the durability of composite restorations, since it is determinant on polymerization characteristics, as degree of conversion [13–15], and poorly cured restoration has lower clinical longevity [16].

Currently the most used photoinitiator in commercial restorative composites is camphorquinone (CQ), a type II Norrish photoinitiator that requires a coinitiator (usually a tertiary amine) to trigger the polymerization reaction [17, 18]. CQ undergoes a hydrogen abstraction photoinitiation: when it absorbs light in wavelength from 430 to 510 nm (blue region of visible light spectra) with maximum absorption wavelength at 468 nm [19, 20], it forms a photoexcitation complex (CQ^*∗*^-amine exciplex) with a tertiary amine (hydrogen-donating agent) generating two free radicals: amino and cetyl. The amino radical is responsible for initiating the polymerization [18]. The great advantage of CQ is its light absorption spectra, that is coincident with emission spectra of the most popular light curing units (LCU) currently used, the blue LED LUCs [19, 21]. Due to this match, the CQ/amine system is able to efficiently initiate the polymerization, fulfilling most of the requirements for an adequate initiation system for a resin-based restorative material. However, some concerns remain about the concentration of the initiator system, especially for flowable composites developed to be used in deep cavities, as they must present great conversion without photoinitiator excess in order to avoid biocompatibility problems [20, 22].

Most available literatures focused mainly on conventional restorative composites, giving limited importance to flowable composites. Although some studies have aimed to evaluate the effect of photoinitiator concentration in higher viscosity restorative composites [10, 13, 23–25], for flowable composites, no study was found. In general, there is some evidence that higher concentrations of photoinitiators can increase the degree of conversion and improve mechanical properties; however, above a certain threshold, no benefits are observed [13]. Also, the intense yellow color of CQ determined by an unbleachable chromophore group limits the quantity of CQ that can be added to an aesthetic restorative material, especially if lighter colors are required [17, 26, 27]. Other concerns refer to biocompatibility; unconsumed CQ may be released [28], producing cytotoxic effect on pulp cells, which is CQ-dose dependent: the higher the concentration, the higher the effect [22].

On the other hand, lower concentration of photoinitiators may have some advantages, once it could delay the development or even reduce shrinkage stress [23], which could favor the maintenance of the interfacial integrity of restorations. In contrast, if concentration of the photoinitiator system was insufficient, a defective polymerization reaction will occur, resulting in poor biocompatibility, unsatisfactory mechanical properties, and chemical instability [20].

Regarding commercial restorative materials, the concentration of photoinitiators is tuned according to monomers present on the resin matrix to modulate the polymerization reaction, since each matrix reacts differently with the photoinitiation system. For this reason, there is a great variability in the concentration of photoinitiators. Taira et al. [29] demonstrated that concentration of CQ varies from 0.17 to 1.03% in weight of the resinous portion of commercially available restorative composites. However, the exact content of the photoinitiator system is not provided by manufactures, making comparison between different materials difficult and speculative. In this way, the use of experimental model materials allows control of composition and better comparison of the evaluated parameters. Using model materials, the real effect of each compound can be defined [5, 10, 12–15]. According to Musanje et al. [13], it is necessary to conduct systematic studies to identify the optimal photoinitiator concentrations, as usage of concentrations beyond the optimal level not only compromises the materials properties but also may impact its overall biocompatibility due to a higher concentration of residual initiator/amine molecules.

Therefore, the aim of this study was to evaluate the effect of photoinitiator concentration on polymerization characteristics and physical-mechanical properties of experimental flowable composites in order to determine the minimum optimal CQ concentration for this kind of composite. Also, it is objective of this study to establish correlations among CQ concentration and degree of conversion, mechanical properties, and color parameters of these materials. The tested hypothesis was as follows: (1) photoinitiator concentration would significantly affect the degree of conversion, mechanical properties, and color parameters of flowable composites; (2) the increase in CQ concentration would increase degree of conversion of composites, mechanical properties, and yellowing, while it would reduce softening rate and luminosity of flowable resin composites.

## 2. Materials and Methods

### 2.1. Formulation of Experimental Composites

All chemicals used in the experimental composites were obtained from Sigma Aldrich (St. Louis, MO 63103, USA). The organic matrix of the experimental composites was prepared using equal parts of the monomers BISGMA (bisphenol A glycerolate dimethacrylate) and TEGDMA (triethylene glycol dimethacrylate), 50 : 50 BISGMA/TEGDMA [12]. The inhibitor BHT (butylated hydroxytoluene) was added to resin the matrix at a concentration of 0.1 wt% in order to prevent spontaneous polymerization of the dimethacrylates. Subsequently, the photoinitiator CQ (Camphorquinone) was added in 5 concentrations (0.25%, 0.50%, 1%, 1.50%, and 2% in weight, considering only the resin matrix). The coinitiator DMAEMA (dimethylamino ethyl methacrylate) was added (photoinitiator/coinitiator ratio of 1 : 2 in weight) [23, 30]. The resin matrix was reinforced at 70% with silanized feldspar fillers (52.5 wt%—Microspar 1351-800 MST, the Mineral Engineers, Frechen—average size: 1 *μ*m) and silanized quartz fillers (17.5 wt%—Silmikron 810-10/1 MTS, the Mineral Engineers, Frechen—average size: 0.5 *μ*m) using a mechanical mixer (DAC 150 SpeedMixer). The composites were prepared in dark room and were kept under refrigerator until one hour before use and then used at room temperature. The formulation of experimental composites were based on previous studies [24, 25, 27].

For all tests performed in this study, experimental composites were light cured with a polywave LED light curing unit (Valo Cordless, Ultradent, South Jordan, USA) with irradiance of 1000 mW/cm^2^ for 20 s, resulting in radiant exposure of 20 J/cm^2^.

### 2.2. Degree of Conversion

The degree of conversion was assessed by Fourier transform infrared spectroscopy (Spectrum One, Perkin Elmer) coupled to an attenuated total reflectance (ATR) device consisting of a diamond crystal of 2 mm in diameter (Diamond ATR, Perkin Elmer). For analysis, 0.05 g of each experimental composite sample (*n* = 5) was directly dispensed onto the diamond crystal and the absorbance spectrum of the unpolymerized condition was obtained. After, the composite was photoactivated as described and the absorbance spectrum of the polymerized condition was obtained. Thirty-two coaddition scans were made at 10 kHz velocity and 4 cm^−1^ resolution. The ratio of the absorbance peak corresponding to the aliphatic carbon-carbon double bond (1637 cm^−1^ peak height) with that of the internal standard, the aromatic carbon-carbon bond (1608 cm^−1^ peak height) of polymerized and unpolymerized condition, was determined [13, 21, 24]. Degree of conversion (DC) was calculated using the follow equation:

(1) $$\begin{array}{c} DC\;\;\left(\;\%\;\right)=100\times \left[\;1-\left(\;\frac{R\text{ polymerized}}{R\text{ non-polymerized}}\;\right)\;\right]. \end{array}$$

*R* represents the ratio between the absorbance bands at 1637 cm^−1^ and 1608 cm^−1^.

### 2.3. Color Analysis

Ten specimens (6 mm diameter × 2 mm thick) of each material were confectioned in a polyvinyl siloxane mold (*n* = 10). The composite was inserted in a single increment, covered with a polyester strip, and light cured as described. After 24 hours of dry and dark storage at 37°C, specimens were submitted to finishing procedures with 600- and 1200-grit SiC papers. Color analysis was performed using a reflectance spectrophotometer (SpectroShade, MICRO, Arbizzano di Negrar, Verona, Italy), calibrated according to manufacturer's recommendations, and equipped with the CIE *L*^*∗*^, *a*^*∗*^, and *b*^*∗*^ system. The value of *L*^*∗*^ is the measure of luminosity or clarity, where total black is *L*^*∗*^ = 0 and total white is *L*^*∗*^ = 100. The axes *a*^*∗*^ and *b*^*∗*^ represent variations of hue and chroma. The axis *a*^*∗*^ measured from reddish (*a*^*∗*^ positive) to greenish (*a*^*∗*^ negative), varying, respectively, from +120 to −120. The axis *b*^*∗*^ measured from yellowish (*b*^*∗*^ positive) to bluish (*b*^*∗*^ negative), varying, respectively, from +120 to −120. Coordinates *a*^*∗*^ and *b*^*∗*^ approximate zero for neutral colors (white, gray) and increase in magnitude for more saturated and intense colors. This system allows the numerical definition of colors, as well as the quantification of differences between them. Thus, for determination of yellowing, the values of the *b*-axis will be considered; the larger the value of *b*, the greater the yellowing [15, 31, 32].

### 2.4. Surface Hardness and Softening Test

Surface hardness was evaluated in the same specimens used in color evaluation (*n* = 10). Knoop hardness number (KHN) was obtained on the irradiated surface of each specimen with an indenter (HMV-2, Shimadzu, Tokyo, Japan), using a 50 g load for 5 s. Three indentations were performed per specimen. After the initial hardness evaluation (*H*1), the specimens were immersed in absolute ethanol for 24 h at room temperature and KHN was evaluated (*H*2), using the same parameters. After immersion, softening rate of the specimens was calculated by the following formula:

(2) $$\begin{array}{c} \text{Softening rate}=100-\left(\;H2\times \frac{100}{H1}\;\right). \end{array}$$

The softening test has been used as an indirect way to evaluate crosslink density based on the assumption that highly crosslinked polymers are more resistant to degradation and solvent uptake, whereas linear polymers present more space and pathways for solvent molecules to diffuse within their structure. Those materials with lower crosslink density will show a higher softening after exposure to organic solvents such as ethanol [24, 27, 33, 34]. Also, the exposure of the polymers to solvent provides some information about the chemical stability of the composite.

### 2.5. Depth of Cure

Five specimens of each experimental composite were prepared in a stainless steel split mold (3 mm diameter × 5 mm deep; *n* = 5). The composite was light cured as described before. Specimens were dark stored at 37°C for 24 hours in a dry environment. The specimens were positioned horizontally and included in acrylic resin (Vipi Flash, Dental Vipi, Pirassununga, SP, Brazil), the specimen/acrylic resin set was grounded with 80-, 320-, 600-, and 1200-grit SiC papers in an automatic polishing machine (Arotec, Cotia, SP, Brazil) in order to expose the central region of the cylinder. Knoop indentations were made across the section of the composite with an indenter (HMV-2, Shimadzu, Tokyo, Japan), using a 50 g load for 5 s. Three readings were performed 20 *μ*m below the surface and at 1, 2, 3, and 4 mm of depth. The Knoop hardness number (KHN) mean value was calculated from the three indentations for each depth. This method has been previously used to achieve depth of cure [24, 35, 36]. KHN ratio was determined among surface and each depth (1 mm, 2 mm, 3 mm, and 4 mm) and a value of at least 0.8 was used to indicate the acceptable depth of cure [37].

### 2.6. Flexural Strength and Elastic Modulus

Ten bar-shaped specimens of each experimental composite were prepared in a polyvinyl siloxane mold (7 mm wide × 2 mm length × 1 mm thickness, *n* = 10). The material was inserted in a single increment and light cured as described. After 24 h of dry and dark storage at 37°C, specimens were finished with 600- and 1200-grit SiC papers and submitted to the three-point bending test in a universal test machine (Instron, Model 3342, Buckinghamshire, England) for evaluation of flexural strength and elastic modulus (test parameters: distance between supports of 5 mm, compressive loading with crosshead speed of 0.5 mm/min). Each specimen size was individually determined with a digital caliper (Mitutoyo, Brazil). Flexural strength and elastic modulus were calculated by the software of the Universal Testing Machine—Bluehill 2 [12, 38, 39].

### 2.7. Shrinkage Stress

Shrinkage stress was performed on a universal test machine (Instron, model 3342, Buckinghamshire, England) in a high compliance system associated with a gauge length transducer [40–43]. In a previous study, with the similar apparatus and specimen dimensions, the calculated values for the compliance of this system were 1.5 × 10^−4 ^mm·N^−1^ [44].

Poly(methyl methacrylate) cylinders (6 mm diameter × 40 mm or 13 m height) were used as substrate for composites. Bonding surfaces were grounded using 600-grit SiC and the adhesive (Scotchbond MP-3M ESPE, St. Paul, USA) was applied and light cured for 10 s. The 40 mm cylinder was attached to the top of the machine and the 13 mm cylinder to the bottom (through a stainless steel device). This device has a hole that allows the LCU tip to be adapted in contact with the base of the cylinder. The composite was inserted between the treated surfaces of the cylinders. The height of the specimen was 2 mm (factor *C* = 1.5, composite volume = 56.52 mm^3^).

After the insertion of the composite, an extensometer (model 2630-101, Instron) was attached to the rods in order to monitor the distance between them during the test and provide feedback to the machine's actuator to reestablish the initial distance. Therefore, the value registered by the load cell corresponded to the force necessary to maintain the initial height of the specimen in opposition to the force exerted by the shrinking composite. In this situation, the deformation of the structures located within the fixation points of the transducer still influences the value registered by the load cell.

The contraction force rate was monitored for 10 minutes from photoactivation. Five specimens were evaluated per group (*n* = 5). Maximum nominal stress was obtained by dividing the maximum contraction force by the sectional area of the specimen.

### 2.8. Statistical Analysis

After being tested for normality by Shapiro–Wilk test, the data obtained from each test were submitted to one-way ANOVA and Tukey's test, with a global significance level of 95% (*α* = 0.05). Regression analyses were performed for all studied properties using photoinitiator concentration and degree of conversion as independent variables. The software Assistat (Campina Grande, Brazil) was used to conduct statistical analysis.

## 3. Results

**Figure 1:**
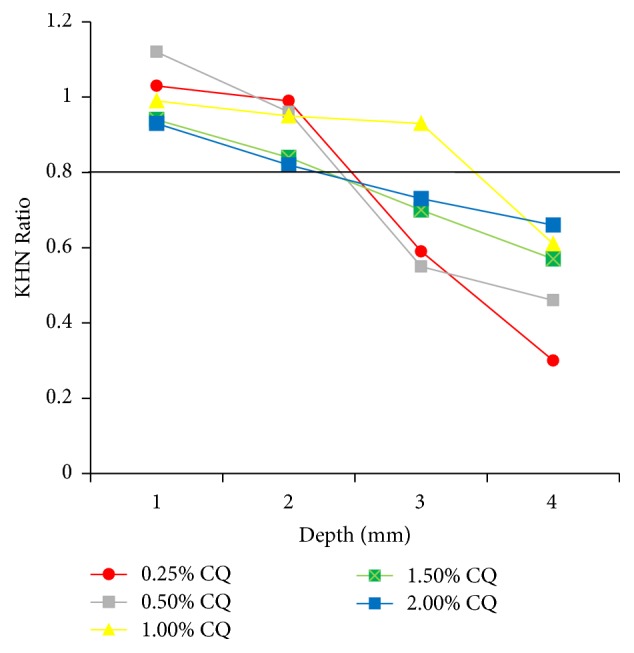
Depth of cure of experimental composites according to concentration of CQ. Black line at 0.8 KHN ratio shows the composites with acceptable ratio. Only the composite containing 1% CQ (yellow line) showed KHN ratio higher than 0.8 at 3 mm depth.

Table 1 shows the mean ± standard deviation of the physical and mechanical properties: degree of conversion (DC), softening rate (SR), surface hardness (*H*), flexural strength (FS), elastic modulus (*E*), shrinkage stress (SS), yellowing (*b*^*∗*^), and luminosity (*L*) of experimental composites per concentration of CQ. The increase in CQ concentration caused a significant increase on degree of conversion and reduction on softening rate, when concentrations of 0.25%, 0.5%, and 1% were compared, while higher concentrations (1.5% and 2%) showed no difference from 1%. Surface hardness was not affected by the concentration of CQ and there was no difference among groups. For flexural strength, the increase on CQ content caused a reduction on mean values; to be precise, composites containing 0.25%, 0.5%, and 1% of CQ showed significant higher flexural strength than composites containing 1.5 and 2%. Elastic modulus and shrinkage stress showed same pattern: composite containing 0.25% of CQ showed lower elastic modulus and lower shrinkage stress when compared to other composites with higher concentration of CQ. Depth of cure reached by experimental composites is showed in Figure 1.

**Figure 2:**
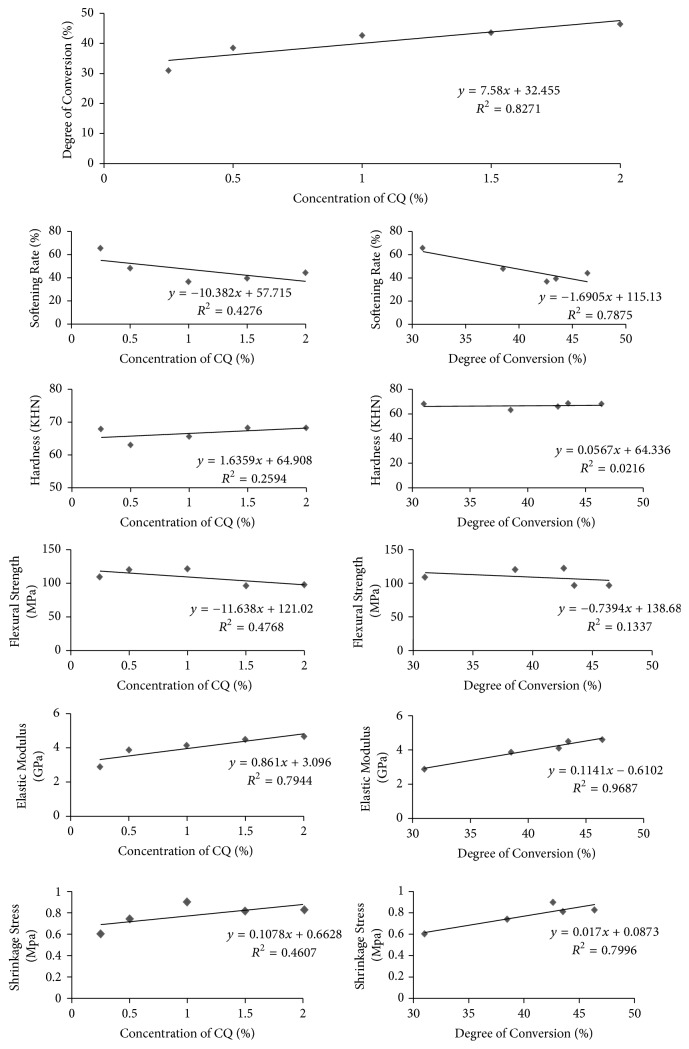
Analysis of linear correlation between concentration of CQ and degree of conversion versus softening rate, surface hardness, flexural strength, elastic modulus, and shrinkage stress.

The correlation coefficients of evaluated properties as a function of concentration of CQ and degree of conversion are shown in Figure 2. The increment in concentration of CQ is directly associated with increase in degree of conversion and elastic modulus, with strong positive correlation (*R*^2^ ≈ 0.8, *p* < 0.05). However, no correlation could be observed between CQ concentration and softening rate, surface hardness, flexural strength, and shrinkage stress (*R*^2^ < 0.5, *p* > 0.05).

Also, it was evaluated the correlation between degree of conversion and mechanical properties (softening rate, surface hardness, flexural strength, elastic modulus, and shrinkage stress). A negative correlation was observed between degree of conversion and softening rate (*R*^2^ ≈ 0.8, *p* < 0.05). The higher the degree of conversion, the lower the softening rate. A strong positive correlation was observed between degree of conversion and elastic modulus and shrinkage stress (*R*^2^ ≈ 0.8, *p* < 0.05). And no correlation was observed between degree of conversion and surface hardness and flexural strength (*R*^2^ < 0.5, *p* > 0.05).

**Figure 3:**
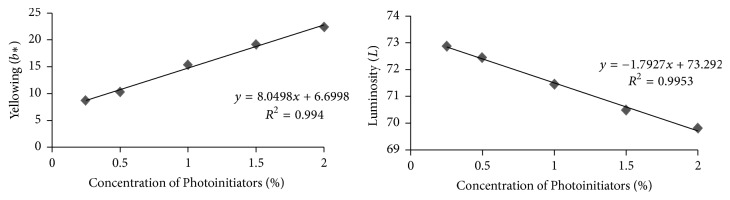
Analysis of linear correlation between concentration of photoinitiators and color parameters (yellowing and luminosity).

Analysis of linear correlation between concentration of CQ and color parameters (yellowing and luminosity) is shown in Figure 3. A strong positive correlation can be observed (*R*^2^ ≈ 1, *p* < 0.05) between CQ concentration and yellowing (*b*^*∗*^) and a strong negative correlation (*R*^2^ ≈ 1, *p* < 0.05) between CQ concentration and luminosity (*L*).

## 4. Discussion

According to the results of this study, most composite curing features are strongly influenced by the amount of photoinitiator within. Thus, the first tested hypothesis was partially accepted: the photoinitiator concentration significantly affected the degree of conversion, mechanical properties, and color parameters of flowable composites, except for surface hardness. The only property that was not affected by photoinitiator concentration was surface hardness. It could be explained by an adequate polymerization in this area in all materials, since the photons for light activation of CQ are completely available and the light intensity provided by the polywave light curing unit is high (1000 mW/cm^2^).

Ideally, the concentration of photoinitiator in resin-based composites systems should be limited to that necessary to promote maximum monomer conversion [13]. It has been reported that there is an ideal level for the increase of CQ concentration, and above this level the increase in photoinitiator does not benefit the final grade conversion [23], which corroborates with the results of the present study. According to our results, the increase in CQ amount until 1% led to a higher level of monomer conversion, reducing the polymer degradation and increasing depth of cure, being the lower concentration with the better properties. According to Table 1, there is no significant difference in degree of conversion, softening rate, surface hardness, elastic modulus, and shrinkage stress when CQ concentrations of 1%, 1.5%, and 2% were compared. Also, concentration of 1% showed higher flexural strength, lower yellowing (*b*^*∗*^ value), and higher luminosity (*L* value) than CQ concentrations of 1.5 and 2%. In this way, second hypothesis cannot be entirely accepted, because although the degree of conversion and most mechanical properties had increased with the increase of CQ concentration, it happened just until 1%, and higher CQ concentrations caused no benefits.

Another advantage of the concentration of 1% CQ in flowable composites was the increase of depth of cure (Figure 1), where the material containing 1% CQ reached 3 mm of depth of cure while others reached only 2 mm. According to Asmusen et al. [17], the presence of light absorbing photoinitiators in resin composites inevitably results in attenuation of the light intensity along the radiation path and often limits the cure depth of these materials [17, 27]. However, when the photoinitiator photobleaches, absorbance decreases and attenuation is reduced since the photoinitiator consumption is accompanied by a deeper penetration of the light through composite thickness [17]. For composites containing higher concentration of CQ, the excess of unreacted photoinitiator reduces photobleaching, not allowing the reduction of the light attenuation; that is why, for composites containing 1.5% and 2% CQ, depth of cure is reduced when compared to composite containing 1% CQ. The reduction in hardness when CQ was increased beyond the optimum concentration of 1% may be also attributed to yellowing of the material due to the high CQ concentration that may have impeded light penetration. The light absorption of the effective wavelength by CQ molecules in the superficial area filters the light being transmitted to deeper layers [45] and may effectively reduce the hardness in this region. Also, it should be considered that a decrease in effective concentration of free radicals can occur as a result of self-annihilation of initiator radicals, which is expected to increase with the higher CQ concentration in the system due to a higher probability of initiator radical collision. This implies that a certain percentage of the total free radicals generated are trapped at their site of production by undergoing self-annihilation instead of contributing to the polymerization process. In other words, a high CQ and amine concentration may result in the generation of very high concentration of free radicals, of which only a fraction may participate in the polymerization reaction [13].

Although the composite contains 0.25% CQ showed reduced shrinkage stress, lower yellowing (lowest *b*^*∗*^ value), and higher luminosity (highest *L* value), which could be considered an advantage; this concentration, as well as 0.5% CQ, was insufficient to promote effective polymerization, since degree of conversion was very low and softening rate is too high. This is in agreement with the results of Meng et al. [20] that also found that 1% of photoinitiators are the minimum concentration required to achieve low cytotoxicity. Results reported by Alonso et al. [25] also support the CQ concentration of 1% for dental restorative composites, since improved marginal and internal adaptations were found for composites containing 1% of CQ when compared to composites containing 0.5% of CQ.

In addition to determine the optimal concentration of photoinitiators in flowable composites, this study also evaluated the correlation between the concentration of CQ and properties of the resin composites and a strong positive correlation (*R*^2^ ≈ 0.8) among CQ concentration and degree of conversion and elastic modulus was found.

The positive correlation among CQ concentration and DC was expected, as higher concentration of photoinitiators increases the probability of polymerization growing centers to be activated [46]. In this way more free radicals can be formed when more CQ/amine molecules are present. This is also in consonance with findings of previous studies [23, 47]. Additionally, the positive correlation between CQ concentration and elastic modulus is probably related to degree of conversion, as the strengthening in elastic modulus occurs with the increase in degree of conversion. No linear correlations among CQ concentration and other tested properties (softening rate, surface hardness, flexural strength, and shrinkage stress; *R*^2^ < 0.5) could be established. Besides, most of these properties are affected by photoinitiator concentration, as seen in Table 1, where there are no linear correlations.

A strong linear correlation was found among CQ concentration and color parameters, positive for yellowing, and negative for luminosity, meaning that the higher the concentration of CQ, the higher the yellowing and the lower the luminosity, corroborating with results of Schneider et al. [27]. It also confirms that the color of a composite is strongly affected by CQ concentration. CQ displays an intense dark yellow color due to the presence of the conjugated diketone chromophore that absorbs at 470 nm. During irradiation of CQ and reduction of one of the carbonyl groups, the conjugation is destroyed, causing a blue shift of the remaining ketone's absorption and loss of the yellow color [17]. This processes is called photobleaching. However, to achieve complete photobleaching, a very long exposure time is required. So, due to incomplete consumption of CQ, composites may remain yellow even after polymerization reaction. Schneider et al. (2009) suggested that the more intense yellow color on polymerized resin composites may indicate the presence of unreacted species. This feature limits the addition of CQ to composites, since it turns them excessively yellow, risking the final esthetic result. Other than producing the undesired yellowing effect, excess photoinitiator and products of their photolysis may leach out from the material into the saliva, with possible cytotoxic effects [48]. Another feature that may contribute to darkening and yellowing of resin composites is the presence of tertiary amine [27]. In this study, the CQ : DMAEMA proportion was 1 : 2 in weight; according to the study of Schneider et al. [27], this ratio is the most adequate since it improved polymer properties but also produces more yellowing in resin composites.

In addition to correlation between CQ concentration and properties of composites, correlation between degree of conversion and properties was also calculated. No correlation was found between degree of conversion and hardness or flexural strength, which disagrees with results founded by Gonçalves et al. [12], who reported a significant correlation among those variables. However, some important methodological differences between these studies can explain this. In Gonçalves et al. study, composites were evaluated 15 minutes after photoactivation and the nonirradiated surface was evaluated [20]. In our study, degree of conversion was evaluated immediately after photoactivation, while surface hardness and flexural strength were evaluated 24 h after photoactivation, which allowed the composites to complete the polymerization reaching the maximum mechanical properties. Other studies also reported lack of correlation among surface hardness and degree of conversion, as Chung and Greener [49] and Bouschlicher et al. [37].

Another important property herein evaluated was shrinkage stress. Shrinkage stress is a local physical condition, not a basic property, and, as such, stress values vary depending on the testing system used, due to differences in geometries, test configurations, and system compliance. An inverse relationship between compliance and shrinkage stress has been described [40–42]. Several studies have shown that the strain capacity of the testing system components has great influence on the stress values. The sum of these deformations is referred to as the system's compliance [40, 42]. The higher the compliance, the higher the system's strain capacity and therefore the lower the recorded stress values [40]. In this study, a high compliance system was used in order to evaluate the materials under stiffness conditions more akin to those found in their clinical use.

In the present study, the lowest shrinkage stress value was recorded for 0.25% CQ, the lowest photoinitiator concentration. The reduction on the stress in for this material can be explained by 2 main reasons: the lower conversion and the reduction in reaction rate with use of low CQ level [23]. CQ concentration would affect polymerization rate: the lower the CQ concentration, the lower the polymerization rate [23]. By slowing the polymerization reaction, stress release can occur, resulting in lower shrinkage stress. According to Braga & Ferracane [50], the effects of reduced curing rates on contraction stress are limited and significant reductions in stress can be verified only after the curing rate drops below a certain threshold. This seems to be the reason for shrinkage of stress data strongly correlated to degree of conversion (*R*^2^ ≈ 0.8) but not correlated to CQ concentration (*R*^2^ < 0.5). The correlation between degree of conversion and shrinkage stress has been established before [50].

## 5. Conclusions

Within the limitations of this study, it is possible to conclude that the photoinitiator concentration affects the degree of conversion, mechanical properties, and color parameters of flowable composites. The increase in concentration of CQ until 1% is directly associated with the augment in conversion degree and elastic modulus of flowable composites. However, although CQ concentration affects the softening rate, flexural strength, and shrinkage stress, there is no linear correlation between these properties and CQ concentration. Moreover, surface hardness is not affected by CQ concentration. Considering the color parameters, the higher the concentration of CQ, the higher the yellowing and the lower the luminosity. The minimum optimal concentration of CQ in BISGMA/TEGDMA flowable composite is 1% wt of the resin matrix, since this CQ concentration allows adequate balance among degree of conversion, depth of cure, mechanical properties, and color characteristics of these materials.

## Figures and Tables

**Table 1:** Degree of conversion (DC), softening rate (SS), surface hardness (*H*) flexural strength (FS), elastic modulus (*E*), shrinkage stress (SS), color parameters (yellowing: *b*^*∗*^ and Luminosity: *L*) of experimental flowable composites according to concentration of CQ. Mean (standard deviation).

[CQ]	DC (%)	SR (%)	*H* (KHN)	FS (MPa)	*E* (GPa)	SS (MPa)	*b* ^*∗*^	*L*
0.25%	31.01 (2.31) c	65.52 (5.71) c	67.84 (7.55) a	109.07 (7.81) a	2.88 (0.54) b	0.60 (0.08) b	8.68 (0.64) a	72.87 (0.57) a
0.50%	38.52 (1.53) b	48.38 (6.25) b	63.05 (8.11) a	120.42 (20.12) a	3.89 (0.95) a	0.74 (0.05) a	10.28 (1.80) a	72.44 (0.48) a
1%	42.63 (1.08) a	36.70 (4.38) a	65.59 (5.63) a	121.15 (17.14) a	4.11 (0.85) a	0.90 (0.14) a	15.30 (4.03) ab	71.45 (0.31) b
1.50%	43.50 (1.62) a	39.44 (4.86) ab	68.47 (5.98) a	96.01 (08.93) b	4.50 (1.08) a	0.81 (0.09) a	19.12 (6.55) bc	70.48 (0.41) c
2%	46.41 (0.94) a	44.03 (8.14) ab	68.18 (5.78) a	97.35 (16.49) b	4.62 (0.75) a	0.83 (0.14) a	22.38 (6,98) c	69.81 (0.35) c

Means followed by the same letter in column indicate lack of significant difference according to Tukey's test.
